# Congenital gastric ectopic pylorus: a rare gastric malformation

**DOI:** 10.1055/a-2333-9480

**Published:** 2024-06-12

**Authors:** Chang Wang, Shengwu Pan, Yaqi Zhai

**Affiliations:** 1 Department of Gastroenterology, Peopleʼs Liberation Army Joint Logistic Support Force 985th Hospital, Taiyuan, China; 2 651943 Division of Gastroenterology and Hepatology, Chinese PLA General Hospital First Medical Center, Beijing, China

A 71-year-old man who presented with hematemesis and melena was admitted to our hospital. He denied any previous surgery history or recent use of alcohol and antithrombotic agents. Esophagogastroduodenoscopy (EGD) revealed an opening leading to the duodenum and the blind antrum without normal pylorus ( Fig. 1 , Fig. 2 ). The ectopic pyloric opening was located in the lesser curvature of the upper gastric body, approximately 2 cm distal to the cardia. Multiple ulcers were also found near the opening without active bleeding ( Video 1 ). The upper gastroenterography and computed tomography showed the teapot-like stomach, further confirming the ectopic pyloric opening in the upper gastric body ( Fig. 3 , Fig. 4 ). With conservative medication, the patient gained a rapid recovery and was discharged uneventfully.

**Fig. 1:**
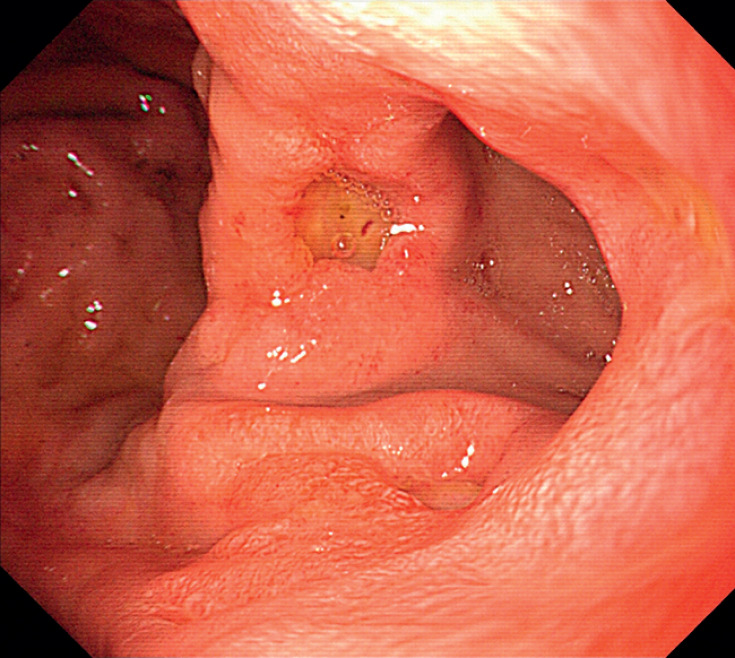
An ectopic pyloric opening leading to the duodenum was located in the lesser curvature of stomach with multiple surrounding ulcers.

**Fig. 2:**
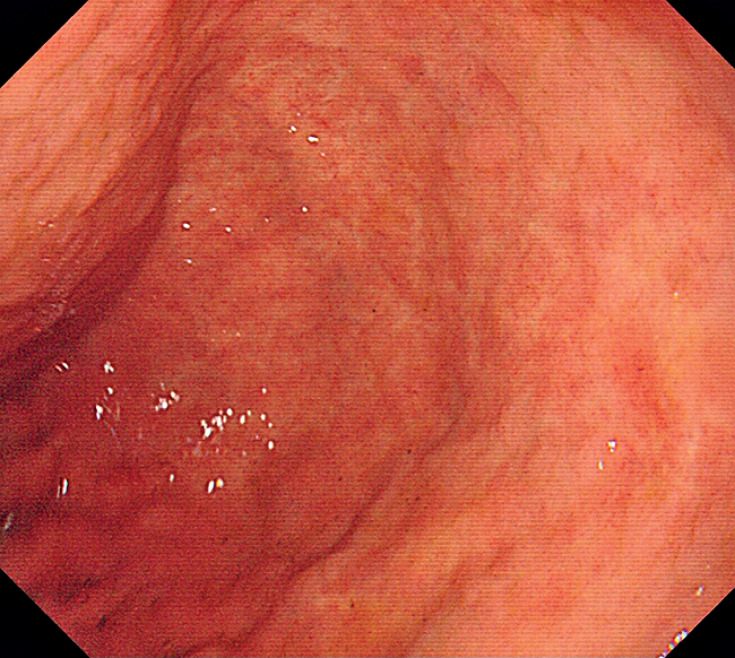
The blind antrum without normal pylorus.

Congenital gastric ectopic pylorus with gastric ulcers.Video 1

**Fig. 3:**
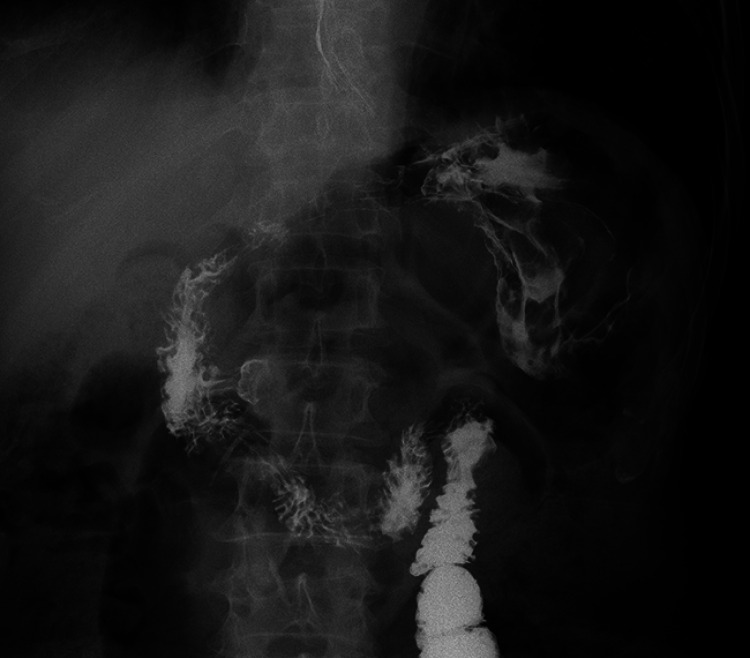
The upper gastroenterography showed the teapot-shaped stomach. The contrast agent accumulated in the blind antrum and flowed from the ectopic opening to the duodenum.

**Fig. 4:**
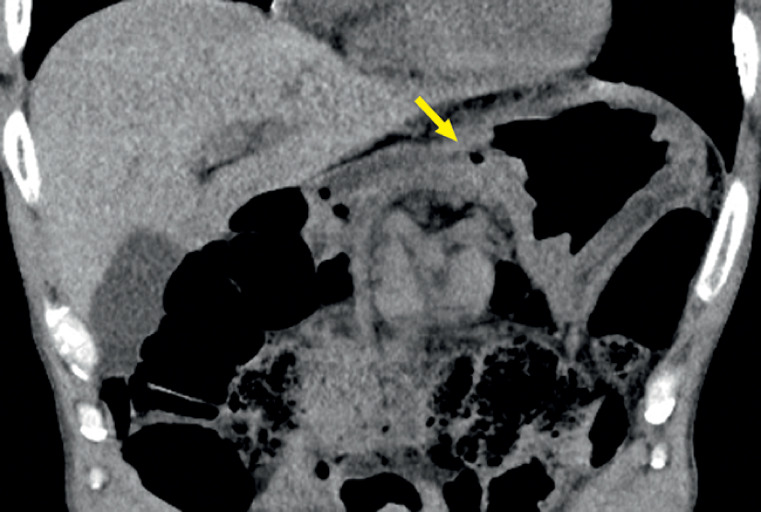
An abdominal computed tomography scan showed the ectopic pylorus opened to the less curvature of stomach (yellow arrow).

Congenital gastric ectopic pylorus is an extremely rare congenital gastric abnormality, which was first reported and coined by Yu ZL et al. 1 in 1983. Uraz S et al. 2 published the first English literature in 2007. This disease occurs more frequently in the 6th and 7th decade of life, with a male predominance (8:1). The majority of reported cases (89.2%) were from East Asia, and the underlying mechanism remained unknown. Most patients present non-specific symptoms, such as abdominal pain, bloating, regurgitation, and belching. However, almost 30% of patients may manifest with upper gastrointestinal bleeding. With continuous acid reflux from the stomach to the duodenum, the constantly open ectopic pylorus may contribute to peptic ulcers and subsequent bleeding 3 . We should improve our understanding of this disease to avoid misdiagnosis.

Endoscopy_UCTN_Code_TTT_1AO_2AB

## Notes

Endoscopy E-Videos https://eref.thieme.de/e-videos
*E-Videos* is an open access online section of the journal *Endoscopy* , reporting on interesting cases and new techniques in gastroenterological endoscopy. All papers include a high-quality video and are published with a Creative Commons CC-BY license. Endoscopy E-Videos qualify for HINARI discounts and waivers and eligibility is automatically checked during the submission process. We grant 100% waivers to articles whose corresponding authors are based in Group A countries and 50% waivers to those who are based in Group B countries as classified by Research4Life (see: https://www.research4life.org/access/eligibility/ ). This section has its own submission website at https://mc.manuscriptcentral.com/e-videos .

## References

[1] Yu ZL Zhao ZY Gastric ectopic pylorus: a case report Chin J Pract Intern Med 1983 3 321

[2] Uraz S Aygün C Konduk T A rare gastric outlet anomaly: pyloric ostium on incissura angularis Dig Dis Sci 2007 52 1001 1003 10.1007/s10620-006-9228-8 17342400

[3] Calhan T Sahin A Kahraman R Atypical placement of the pylorus: a rare congenital abnormality Endoscopy 2014 46 E302 E303 10.1055/s-0034-1377214 25058823

